# Minor Neurological Dysfunctions (MNDs) in Autistic Children without Intellectual Disability

**DOI:** 10.3390/jcm7040079

**Published:** 2018-04-12

**Authors:** Gabriele Tripi, Sylvie Roux, Marco Carotenuto, Frédérique Bonnet-Brilhault, Michele Roccella

**Affiliations:** 1 Dipartment PROSAMI, Paolo Giaccone Hospital, University of Palermo, 90127 Palermo, Italy; 2 Childhood Psychiatric Service for Neurodevelopmentals Disorders, Centre Hospitalier du Chinonais, 37500 Saint-Benoît-la-Forêt, France; 3 UMR 1253, iBrain, Université de Tours, Inserm, 37000 Tours, France; sylvie.roux@univ-tours.fr (S.R.); frederique.brilhault@univ-tours.fr (F.B.-B.); 4 Clinic of Child and Adolescent Neuropsychiatry, Department of Mental and Physical Health, and Preventive Medicine, University of Campania “Luigi Vanvitelli”, 80138Naples, Italy; Marco.CAROTENUTO@unicampania.it; 5 Department of Psychological Sciences, Pedagogical and Education, University of Palermo, 90128 Palermo, Italy; michele.roccella@unipa.it

**Keywords:** minor neurological dysfunctions, autism spectrum disorder, sensory-motor impairment

## Abstract

Background: Children with autism spectrum disorder (ASD) require neurological evaluation to detect sensory-motor impairment. This will improve understanding of brain function in children with ASD, in terms of minor neurological dysfunctions (MNDs). Methods: We compared 32 ASD children without intellectual disability (IQ ≥ 70) with 32 healthy controls. A standardized and age-specific neurological examination according to Touwen was used to detect the presence of MNDs. Particular attention was paid to severity and type of MNDs. Results: Children with ASD had significantly higher rates of MNDs compared to controls (96.9% versus 15.6%): 81.3% had simple MNDs (*p* < 0.0001) and 15.6% had complex MNDs (*p* = 0.053). The prevalence of MNDs in the ASD group was significantly higher (*p* < 0.0001) than controls. With respect to specific types of MNDs, children with ASD showed a wide range of fine manipulative disability, sensory deficits and choreiform dyskinesia. We also found an excess of associated movements and anomalies in coordination and balance. Conclusions: Results replicate previous findings which found delays in sensory-motor behavior in ASD pointing towards a role for prenatal, natal and neonatal risk factors in the neurodevelopmental theory of autism.

## 1. Introduction

Autism and Autism Spectrum Disorder (ASD) describe a range of clinical profiles, which share two main dimensions: social and communication impairment and repetitive behaviors or restricted interests, present during childhood.

These qualitative abnormalities are included in diagnostic criteria used in international classification systems: the American Psychiatric Association Manual of Psychiatric Diseases, 5th Edition (DSM-5) and the World Health Organization’s (WHO) International Classification of Diseases, 10th edition (ICD-10). 

In recent years, attention has focused upon sensory-motor abnormalities observed in subjects with ASD, categorizing these as ‘associated symptoms’. Infants and children with ASD have been found to display altered motor development, clumsiness, retention of primitive reflexes, deficits in gross and fine motor movement, impaired postural control and abnormal gait sequencing [1,2]. Structural and functional neuro-imaging studies, as well as postmortem examinations, suggest a broad area of disordered neuronal organization and cortical connectivity across individuals with ASD. Motor impairments may indicate disruption to fronto-striatal pathways and basal ganglia, as well as alterations in the cerebellar region and brain stem [2,3,4].

Neurological examination is a valuable diagnostic tool for children with sensory-motor problems, as it provides insight into the neurobiological basis and the aetiology of sensory-motor impairment.

One instrument used to assess a child’s neurological condition, with satisfactory reliability [5], is the neurological examination according to Touwen [6]. This is a standardized and age-specific examination, which focuses on minor neurological dysfunctions (MNDs). These dysfunctions have neurodevelopmental relevance, indicating specific alterations in the connecting fiber systems of the central nervous system which result in impaired sensory-motor performance. An adverse event at any stage of neurological development may result in various clinical manifestations of MNDs, at different life stages [7,8]. Two etiologically and clinically different forms of MNDs can be distinguished: simple and complex MNDs. Simple MND may be regarded as an expression of typical, but non-optimal brain wiring. It is moderately related to genetic constitution and prenatal, natal and neonatal adversities, and associated with risk of behavioral problems. In contrast, complex MND denotes clinically relevant brain dysfunctions and is considered to be a borderline form of cerebral palsy [7]. The aim of the present study was to evaluate the neurological condition of ASD children, to investigate presence and topological profile of MNDs as neurological markers of impaired sensory-motor performance in ASD children.

## 2. Experimental Section

### 2.1. Subjects

The current study compared 32 ASD children (28 boys mean age = 8 years 11 months; SD = 3.2) with 32 healthy control children (28 boys; mean age 9 years; SD = 3.1) of similar ages (Student T test; T = −0.10, df 62, *p* = 0.918). The subjects were selected at Tours University Hospital (Tours, France) and at Paolo Giaccone Hospital (Palermo, Italy). An extensive multidisciplinary child neuro-psychiatric assessment was carried out for each subject. Assessment consisted of preliminary developmental history, medical examination and neuropsychological assessment. Preschool children and subjects who had entered into puberty were excluded from the study. Puberty was defined by the presence of secondary sexual characteristics, as suggested by Coleman [9]. The ASD group was selected to include subjects reflecting the symptomatic diagnostic criteria identified by ICD-10 (F84.x). Diagnoses were further evaluated by interviewing parents using the ADI-R [10]. In the routine course of evaluation, all autistic subjects were rated using the Childhood Autism Rating Scale (CARS) [11]; the average score for CARS was 26.9 (SD = 5.1). The ASD group was composed of 18 subjects with Autistic Disorder (AD, F84.0), 7 subjects with Asperger Syndrome (AS, F84.5), 7 subjects with Pervasive Developmental Disorders not otherwise specified (PDD−NOS, F84.8/F84.9). Level of intellectual functioning in ASD group was evaluated using standardized tests including the Wechsler Intelligence Scale for Children III [12] and the “Echelles Differentielles d’Efficience Intellectuelle—Forme Revisée” [13]. Children with intellectual disability were excluded (ICD-10, F70–F79), the mean global Intellectual Quotient (IQ) of the 32 ASD patients was thus 89.6 (SD 14.0). ASD children with motor disabilities of lesional or accidental origin, or, caused by confirmed genetic or neurological disease were excluded. The control group consisted of children with normal psychomotor and language development, regular school attendance and normal scholar level at preliminary developmental history, needing any further clinical investigation for intellectual disability by ICD-10, (F70–F79). Informed consent was obtained from all parents of each participant.

### 2.2. Assessment of Minor Neurological Dysfunctions

As a part of the extensive multidisciplinary child psychiatric assessment, all children underwent a standardized and detailed neurological examination (see Appendix A) according to Touwen [6]. The following clusters were evaluated: posture and muscle tonus; reflexes; involuntary movements; coordination and balance; fine motor movements; associated movements; sensory functions and cranial nerve function.

The assessment was conducted by the same author (GT), across two hospital sites, in the same testing rooms each time, over a duration of 1 h. The assessment criteria used to identify MNDs was age specific; as developmental changes in the nervous system are known to induce changes in the expression and prevalence of MNDs. Literature data [14] indicate that the rate of MNDs at preschool age in the general population is relatively low, reaching its peak before the emergence of puberty. Puberty is associated with a substantial decline in the number of dysfunctional clusters of MND, therefore subjects who had entered puberty were excluded from the study. Signs of dysfunction are taken into account only if they occur in cluster. The presence of a single sign of dysfunction, such as isolated positive Babinski signs, does not lead to a diagnosis of MND. Clusters, as defined in clinical practice, are organized according to the functional neuro-behavioral subsystems of the nervous system [5].

The original protocol suggested by Touwen [6] was adapted slightly. Adaptation consisted of removal of: following an object with rotation of the trunk whilst sitting, palmo-mental reflex, Mayer and Leri reflexes, cremasteric reflex, Galant response, examination of the spine whilst the child is lying, examination of the hip joints, sitting up without the help of hands, fundoscopy and localization of sound. The items were omitted as some authors [15] suggest they have little value in terms of determining the presence or absence of clusters of MND in the general population.

On the basis of the Touwen examination, children were classified as neurologically normal, simple MND, complex MND, or neurologically abnormal. Neurological abnormality (e.g., cerebral-palsy, muscular dystrophy or evidence of other frank neurological pathology) found during evaluation, was an exclusion criteria. Children were classified as neurologically normal if no clusters of dysfunction were present, as having simple MND if one or two clusters of dysfunction were present, and as having complex MND if three or more dysfunctional clusters were present [14,16].

### 2.3. Statistical Analysis

Statistical analyses were conducted using Statistica software (Statsoft, Inc., Tulsa, OK, USA). Descriptive analyses were used to summarize the profile of the study population. Nonparametric statistical analyses (Fisher’s exact probability test) were used to analyze differences between groups. When the result was statistically significant, effect size for proportions (Cohen’s h) was reported to assess the strength of the relationship. Tests were performed on the 2-sided 5% level of significance.

## 3. Results

The prevalence of MND in ASD children was 96.9%, significantly higher than in the control group (15.6%, *p* < 0.0001). The prevalence of simple MND (81.3%) was significantly higher in the ASD group than in the control group (*p* < 0.0001); interestingly, in children with ASD, complex MND (15.6%) occurred more frequently than in non ASD children (0%), with a difference that approached significance (*p* = 0.053) (Table 1).

One of the 32 ASD children was neurologically normal (PDD−NOS, *n* = 1), 26 children had simple MND (AD, *n* = 16; AS, *n* = 5; PDD−NOS, *n* = 5), and 5 children had complex MND (AD, *n* = 1; AS, *n* = 2; PDD−NOS, *n* = 2).

Most dysfunctions except posture and muscle tone regulation, cranial nerve dysfunction and reflex abnormalities occurred more often in children with ASD than in controls, with large or medium effect sizes (Cohen’s *h* > 0.60; Table 2). The most frequent MND was associated movements (59.4%). Mild dysfunction in coordination and balance was present in 34.4% of ASD children and fine motor disturbances in 28.1%. We also found more frequent sensory deficits (21.9%) and involuntary movements (21.9%). Deviances were also found in posture and muscle tone regulation and cranial nerve dysfunction, but less frequently. Non-significant differences were found in MND; this merits further investigation due to the relatively small number of cases in the sample.

## 4. Discussion

This study investigated the prevalence of MND in a group of children with ASD, using a standardized, reliable and age-specific neurological examination (see Appendix A) according to Touwen [6]. We selected only ASD children who had IQs in the normal range (≥70) due to previous evidence that several tasks; tests of graphesthesia, stereognosis, alternating movements, and sequential movements; are unsuitable for use in ASD children with intellectual disability [15,17]. Conversely, Robinson et al. [18] reported that impairments in motor function such as difficulties in planning, inhibition of prepotent responses and self-monitoring, reflect characteristic features that are independent of IQ. A minor limitation of the current study, was that the Touwen examination [6] was carried out by a single pediatric neurologist with longstanding experience in the field of autism.

The current study shows that neurological examination (see appendix) according to Touwen [6] may differentiate the neurological make-up of ASD children from normal children. We also found more frequent occurrence of simple MND and higher prevalence of complex MND which approached statistical significance. Previous literature [8] suggests that simple MND might be largely (epi)genetically determined or mildly associated with prenatal and natal risk factors, such as preterm birth, severe IUGR and mild-to moderate degrees of perinatal asphyxia. Postnatal infections and sepsis are also frequently considered in literature as the most important risk factor for developing simple MND. Similar chains of prenatal, natal and neonatal adversities are known to play a strong role in the etiology of complex MND, suggesting that it might be attributed to a lesion of the brain in early development [16]. Interestingly, in a recent study [19] the evaluation by transcranial ultrasonography of ASD children and neurotypical siblings show relevant findings of cortical dysplastic lesions in the ASD cohort. The current results are consistent with previous research [20], highlighting frequent association between unfavorable prenatal, natal and neonatal events in pregnancy, and autism.

With respect to specific MNDs, our study found a wide range of fine manipulative disability, sensory deficits and choreiform dyskinesia in the ASD group. Several studies examining subgroups of MND have reported that behavioral problems might be more likely to occur in conjunction with fine manipulation, hypotonia, and choreiform dyskinesia [8]. In the general population, fine manipulation disability has been frequently related, with dysfunctional cortico-striato-thalamo-cortical pathways [21]. Furthermore, a substantial proportion of ASD children show sensory dysfunction, reflecting anomalies in the structure of thalamic nuclei, which represents a crossroads of sub-cortical afferences and efferences toward the pre-motor associative zones, with an integrating role for sensory pathways [3,22,23].

In our study, almost 2/3 of ASD children showed an excess of associated movements and over 1/3 of ASD children showed mild dysfunction in coordination and balance. These motor anomalies are caused by dysfunction in fronto-striatal pathways [24,25] and cerebello-thalamo-cortical pathways respectively [7] and result in executive function deficit. Results are consistent with previous literature, which found difficulties in diadochokinesis, limb coordination and static/dynamic balance in high-functioning autism and Asperger disorders [26]. The absence of anomalies in deep tendon reflexes and low rating of cranial nerve dysfunction in ASD children, is in line with results of previous studies [25].

Our study indicates a weak association between intellectual disability in ASD and dysfunctional posture and muscle tone regulation. These are often associated with ASD in the literature [2]. However, the results of Ming et al. [4] and Akshoomoff et al. [19] showed that abnormality in muscle tone occurred more commonly in ASD children younger than mean age of our ASD cohort. Due to a relatively small sample size, the current study design did not include evaluation of specific dysfunctions according to age. Interestingly, some authors suggest that mild dysfunctions in muscle tone regulation are not related to prenatal, natal and neonatal events and only show a weak association with behavioral problems [5,7]. Future research into MNDs in ASD children should aim to correlate specific MNDs with age groups, as well as prenatal, natal and neonatal characteristics.

In the present study, no neuroimaging data of the ASD children were available, but the results of our study add to findings from existing neuroimaging data describing abnormalities in basal ganglia, thalamus, supplementary motor areas and cerebellum as a likely source of sensory-motor impairment in autism [3,5,27]. Further study on the neurological make-up in ASD subtypes, with high and low cognitive function, may shed light on whether motor impairment reflects the severity of brain dysfunction.

In conclusion, this study replicated previously identified anomalies in sensory-motor behavior in ASD children without intellectual disability, through evaluation of neurological condition. The clinical features of sensory-motor impairment point towards a role of prenatal, natal and neonatal risk factors in the neurodevelopmental theory of autism.

## Figures and Tables

**Table 1:** Prevalence of MND in ASD and control children.

Neurological classification	ASD (*n* = 32) *n* (%)	Control (*n* = 32) *n* (%)	*p* Value	Cohen’s *h*
Normal	1 (3.1)	27 (84.4)	*p* < 0.0001	1.97
Simple MND	26 (81.3)	5 (15.6)	*p* < 0.0001	1.43
Complex MND	5 (15.6)	0 (0)	*p* = 0.053	0.81

MND-minor neurological dysfunctions; ASD- autism spectrum disorder.

**Table 2:** Prevalence of the specific types of MND in ASD and control children.

Type of MND	ASD (*n* = 32) *n* (%)	Control (*n* = 32) *n* (%)	*p* Value	Cohen’s *h*
Posture and muscle tone	4 (12.5)	0 (0)	*p* = 0.113	--
Reflex abnormalities	0 (0)	0 (0)	--	--
Involuntary movements	7 (21.9)	0 (0)	*p* = 0.011	0.97
Coordination and balance	11 (34.4)	3 (9.4)	*p* = 0.032	0.63
Fine motor dysfunction	9 (28.1)	2 (6.3)	*p* = 0.043	0.61
Associated movements	19 (59.4)	1 (3.1)	*p* < 0.0001	1.41
Sensory deficits	7 (21.9)	0 (0)	*p* = 0.011	0.97
Cranial nerve dysfunction	1 (3.1)	0 (0)	*p* = 1.0	--

MND-minor neurological dysfunctions; ASD- autism spectrum disorder.

## Appendix A. Clusters of Neurological Dysfunctions

**Table:**

Cluster of Dysfunction	Based on	Criteria for Dysfunctional Cluster
Dysfunctional muscle tone regulation	- Muscle tone - Posture during sitting, crawling, standing and walking	One ore more of the following: - mild deviations of muscle tone in legs - mild deviations of muscle tone in arms - consistent mild deviations in posture
Reflex abnormalities	Intensity tendon reflexes arms: high, low or asymmetrical: - Threshold tendon reflexes arms: high, low or asymmetrical - Intensity tendon reflexes legs: high, low or asymmetrical - Threshold tendon reflexes legs: high, low or asymmetrical - Foot-sole response: uni- or bilateral Babinski sign - Plantar grasp: uni- or bilaterally present - Abdominal skin reflex: asymmetry	Presence of at least two signs
Involuntary movements	Spontaneous motor behavior: - Test with extended arms - Movements of face, eyes, tongue	Presence of at least one of the following: - marked, consistent choreiform movements of distal muscles - marked, consistent choreiform movements of proximal muscles - marked choreiform movements of face, eyes and/or tongue - marked, consistent tremor - consistent athetotiform movements in distal muscles
Coordination and balance	- Finger-nose test - Fingertip-touching test - Diadochokinesis - Kicking - Knee-hell test - Reaction to push (sitting, standing) - Romberg - Tandem gait - Standing on one leg - Hopping on one leg	Presence of age inadequate performance of at least three tests
Fine manipulative ability	- Finger opposition test: smoothness - Finger opposition test: transition - Finger–tip test - Circle test - Taping-with-pencil test	Two or more tests inappropriate for age
Associated mouvements	Associated movements during: - Diadochokinesis - Finger opposition test - Walking on toes - Walking on heels	Presence of an excessive amount of associated movements for age in at last two tests
Sensory deficits	- Mouth-opening-finger-spreading phenomenon - Graphesthesia - Kinaesthesia - Sense of position - Hearing - Visual fields	Two or more sensory functions dysfunctional
Cranial nerve dysfunction	- Motor behaviour of face, eyes, pharynx and tongue	Mild cranial nerve palsy
